# Toward an indoor lighting solution for social jet lag

**DOI:** 10.21203/rs.3.rs-2649098/v1

**Published:** 2023-03-17

**Authors:** Alex Neitz, Alicia Rice, Leandro Casiraghi, Ivana L. Bussi, Ethan D. Buhr, Maureen Neitz, Jay Neitz, Horacio O. de la Iglesia, James A. Kuchenbecker

**Affiliations:** 1 Department of Biology and The Molecular and Cellular Biology graduate program, University of Washington, Seattle, Washington, USA; 2 Department of Biology, University of Washington, Seattle, Washington, USA; 3 Department of Ophthalmology, University of Washington, Seattle, Washington, USA

## Abstract

There is growing interest in developing artificial lighting that stimulates intrinsically photosensitive retinal ganglion cells (ipRGCs) to entrain circadian rhythms to improve mood, sleep, and health. Efforts have focused on stimulating the intrinsic photopigment, melanopsin; however, recently, specialized color vision circuits have been elucidated in the primate retina that transmit blue-yellow cone-opponent signals to ipRGCs. We designed a light that stimulates color-opponent inputs to ipRGCs by temporally alternating short and longer wavelength components that strongly modulate short-wavelength sensitive (S) cones. Two-hour exposure to this S-cone modulating light produced an average circadian phase advance of one hour and twenty minutes in 6 subjects (mean age = 30 years) compared to no phase advance for the subjects after exposure to a 500-lux white light equated for melanopsin effectiveness. These results are promising for developing artificial lighting that is highly effective in controlling circadian rhythms by invisibly modulating cone-opponent circuits.

## Introduction

**Figure 1.**
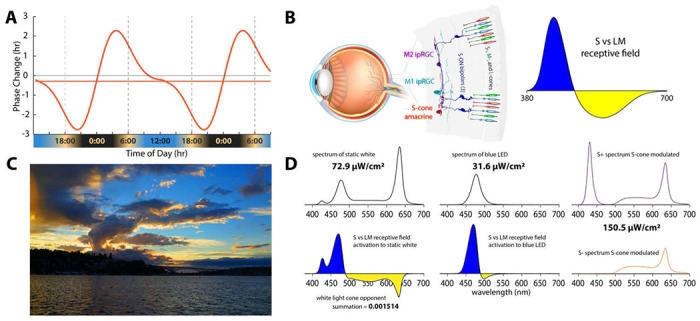
**A.** Phase response curve based on Khalsa (2003)^5^ that is aligned with earth time so that the beginning of the internal biological night occurs at sunset and the end of the internal biological night occurs before wake time just after sunrise as indicated below the x-axis of the curve. **B.** (left) Illustration of the color vision circuitry for S-ON and S-OFF types of primate ipRGCs. (right) Illustration of the spectrally opponent response of an S-ON ipRGC with S - (L+M) cone inputs. **C.** Image of sunset in Seattle Washington illustrating how contrasting short and long wavelength light near the horizon produce a stimulus capable of driving spectrally opponent inputs to ipRGCs making them act as sunrise/sunset detectors. **D.** Spectral distributions of experimental light stimuli and their predicted effects on the color opponent inputs to ipRGCs. (Top left) Spectrum of the experimental white light with chromaticity coordinates 0.333, 0.333. (Top middle) Spectrum of the LED-derived experimental “blue” light with a spectral peak at 476 nm. (Bottom; left and middle) the product of wavelength-by-wavelength multiplication of the spectral distribution of the white light (Bottom left) times the spectrally opponent response of an ipRGC. Integration of the curve in across wavelength yields the predicted very small relative response of the ipRGC to the white light. (Bottom middle) The product of multiplication of the spectral distribution of the blue light times the spectrally opponent response of an ipRGC. Integration across wavelengths yields the predicted large relative response of the ipRGC to the blue light. (Right) The two spectra which are alternate to produce the S-cone modulating light.

People who spend most of their time under artificial light often suffer a phase delayed circadian rhythm^1–3^. The discrepancy between an individual’s delayed biological rhythm and the daily timing determined by social constraints like school and work schedules causes “social jet lag”^4^ which is associated with disturbed sleep, daytime fatigue, reduced cognitive function, and a general feeling of unwellness. A potential solution to social jet lag is to develop artificial lighting that is capable of stimulating ipRGCs in the morning during times when such stimuli produce phase advances^5^ (Figure 1A). With regard to circadian rhythms there has been an emphasis the effects of light on the intrinsic photopigment, melanopsin, however ipRGCs can be activated by light absorption by cone photoreceptors whose signals are carried by color opponent circuitry (Figure 1B) in which short (S) and long (L) plus middle (M) wavelength cones have opposite signs ^6–8^. The color opponent input to ipRGCs may have evolved so that changes in the color of sky at dawn and dusk (Figure 1C) can contribute to synchronization of the internal circadian clock such that the internal biological night begins at sunset and ends before wake time just after sunrise. Previous experiments have provided evidence for a role for color opponency in circadian phototransduction^9^ and clear evidence for an S-cone contribution in humans^10,11^.

Compared to melanopsin, cone-opponent circuits activate ipRGCs at much lower thresholds^12^. Thus, at common indoor low illumination levels, lights optimized to stimulate the color-opponent circuits could be much more effective in producing circadian phase advances than typical white artificial lighting. Color opponent circuitry in humans is normalized through experience to null to white^13^. Thus, even though artificial white light stimulates S-cones, because the excitatory and inhibitory cone components of the S vs. (L+M) circuitry are balanced by white light it is predicted to have little net effect (Figure 1D). Narrowband lights that primarily stimulate one side of the opponent circuit are predicted to be much more effective (Figure 1D). Finally, the circuity carrying cone signals has relatively transient response properties, so under laboratory conditions using narrow band lights that primarily stimulate S-cones, their contributions decay upon extended light exposure^10,11^. Thus, the intensity, spectral and temporal characteristics of the light must all be considered when developing indoor illumination capable of combating social jet lag.

We designed a light that stimulates color-opponent inputs to ipRGCs by temporally alternating short and longer wavelength components that strongly modulate short-wavelength sensitive (S) cones. We determined the ability of a morning exposure of this light to produce a phase advance capable of combatting social jet lag compared to a static white light and a static narrow band blue light. Our goal is to evaluate the most effective dynamic lighting approach for circadian photoentrainment at the comparatively low general lighting lux levels typical for homes, offices, schools, and health care facilities. We hypothesize that practical lighting solutions that drive cone-based color-opponent inputs to ipRGCs in the early morning can mediate circadian phase advances that will promote improved mood and cognitive function, combat social jet lag and other circadian problems such as seasonal affective disorder.

## Results

### Participants circadian phase relative to solar time

**Figure 2.**
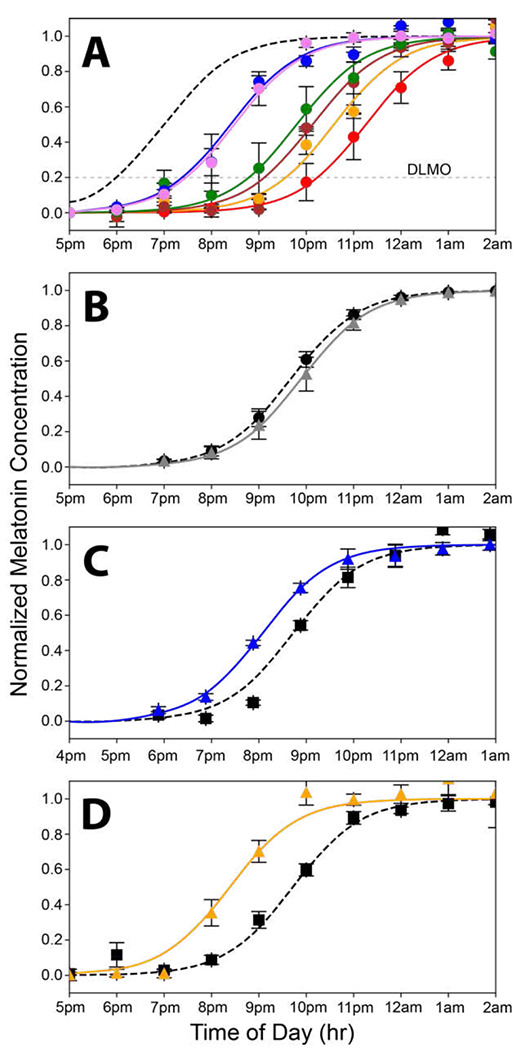
Curves showing the nighttime dim rise in salivary melatonin levels under various conditions equated for melanopsin effectiveness. **A.** Rise in evening melatonin levels for the six subjects who participated in this study (each is shown in a different color). The dashed gray curve shows the predicted rise if the subjects were aligned to earth time where beginning of internal biological night occurs at sunset. On average, subjects were phase delayed 2.8 h. **B.** Average rise in evening melatonin after two-hour exposure to the static white light (gray curve) of Figure 1A compared to a baseline (dashed curve) measured on day one of the 3-day protocol. There was a slight, nonsignificant, phase delay associated with the white light exposure (n=3 subjects). **C.** Average rise in evening melatonin (blue curve) after a two-hour exposure to the 476-nm blue light of Figure 1B compared to baseline (dashed curve) (n=6 subjects). The 476-nm light produced a phase advance of 40 minutes. **D.** The Rise in evening melatonin (orange curve) after two-hour exposure to 19 Hz S-cone modulated light compared to baseline (dashed curve) (n=6 subjects). This light produced a phase advance of 1 hour and 20 minutes.

When humans are exposed only to natural light, the internal circadian clock synchronizes to solar time such that the internal biological night begins at sunset and ends before wake time just after sunrise^1^ (Figure 1A). We used dim light salivary melatonin onset (DLMO) as a measure of circadian phase. Figure 2A shows the rise in evening melatonin levels assayed from saliva samples for the six subjects who participated in this study (each subject is represented by a different color). Compared to being synchronized to solar time (shown by the dashed gray curve; Figure 2A), we found that, on average, subjects were phase delayed by 2.8 hours. Baseline results for each subject were comparable across multiple days, indicating that while all subjects were phase delayed, they were still entrained to the 24-hour environmental cycle. These phase delay results are like those observed earlier for young academics^1^, highlighting the need for an artificial lighting solution that can effectively bring the sunlight indoors. Later chronotypes are associated with longer phase delays^1^ consistent with the considerable variability in the phase delays among the six subjects.

### Spectral and temporal characteristics of lights capable of producing phase advances

We measured the effectiveness of lights with different spectral and temporal properties in generating circadian phase advances. Lights were calibrated to produce the same time-averaged effect on melanopsin, but to have large differences in their effect on the recently discovered color vision circuits that drive M1 and M2 ipRGCs ^7,8^, as illustrated in Figure 1B. One light had the same CIE coordinates (x = y = 0.33) as an equal energy white and produced an illuminance of 500 lux (Figure 1D; top left). A second light was blue, derived from an LED (476 nm peak) (Figure 1D top right). These two lights are predicted to have very different effects on the color vision circuits carrying chromatically opponent signals from short (S-), middle (M-), and long (L-) wavelength sensitive cones that drive ipRGCs in primates ^7,8^ (Figure 1D bottom right and left). Primate ipRGCs display an S vs. (L+M) color-opponent spectral response (Figure 1B right). The white light drives both the excitatory and inhibitory sides of the color-opponent response, thus producing little net drive to the ipRGCs from cones. In contrast, almost all wavelengths in the blue light stimulate the S-cones on the excitatory side of the response of the color-opponent system. Thus, the white light is expected to produce a null response, and the blue light is predicted to be many times more effective at driving the color-opponent pathways upstream of the ipRGCs (Figure 1D).

To evaluate the ability of lights with different spectral and temporal characteristics to advance circadian phase, we followed a 3-day protocol for each light condition. On the evening of the first day, subjects collected saliva samples every hour starting at 6 PM ending at 2 AM. The following day, the samples were analyzed to measure the rise in melatonin the evening before and the time of DLMO was determined for each subject, defined as the time the melatonin levels reached 20% of maximum^14^. On the morning of the third day of the protocol, each subject viewed a test light for two hours centered 10.5 hours after their individual DLMO. This corresponds to the time of circadian cycle expected to produce the maximum light-induced phase advance (Figure 1A)^5^. On the evening of the same day, subjects again collected saliva samples that were used to evaluate whether the light exposure produced a phase advance.

Figure 2B shows the results for the static white light. After exposure to the static white light, the average rise in evening salivary melatonin levels did not differ significantly from the baseline, measured before exposure). The slight phase delay after the exposure is within experimental error (p<0.05; paired t-test). In contrast, the 470 nm blue light that was equated in melanopsin effectiveness to the static white light produced a phase advance of 40 minutes (Figure 2C).

Our goal is to develop lighting that can replace standard indoor white lighting and give people control of their circadian phase. A static blue light (like Figure 1D; top left) is not an acceptable substitute for standard lighting because it must be pure blue to drive the color vision circuitry. Any added long-wavelength components that make the light whiter, cancel the effectiveness. As an alternative, we tested a temporally modulated light because, unlike the melanopsin drive to ipRGCs, which is quite sustained, the cone inputs have transient responses. There are two types of color-opponent ipRGCs in primates, S-ON and S-OFF, but both are ON-OFF cells responding both to the onset of one colored light and the offset of the light of the opposing complementary color^6^.

Thus, theoretically, the best stimulus is a light that alternates between short and long-wavelength components such that the color-opponent cells are being stimulated by the simultaneous offset of one spectral component and the onset of the opposing component. It is possible to produce lights that, when temporally alternated, appear white but strongly modulate S-cones. The S-cone inputs to ipRGCs are tuned to respond to higher temporal frequencies than those serving hue perception making it possible to modulate the S-cone input to ipRGCs strongly but minimize (and ideally eliminate) the percept of flicker. The S-cone modulating light tested here consisted of a 19 Hz alternating pulse train designed to modulate the quantal catch of S-cones with a differential of 100X between the two phases. This was done by alternating the intensities of LEDs peaking at 427 nm vs. 545 nm, and the addition of light from a 638 nm LED made the S-cone modulated pulse train appear nominally white. The intensity of this light was adjusted to produce a time-averaged quantal catch in melanopsin matched to the 500-lux static white light of Figure 1D. As shown in Figure 2D, the S-cone modulated “white light” elicited a striking 1 hr 20 min phase advance.

## Discussion

Blue lights are particularly effective in driving ipRGCs^15,16^, and it is often assumed this is mediated by melanopsin. However, one novel aspect of the experiments here is that the blue and white lights were equated for melanopsin effectiveness, thus, the large effect of blue compared to white cannot be attributed to activation of melanopsin. Since the white condition nulls the color-opponent response (Figure 1D; left), it effectively isolated the melanopsin drive to the ipRGCs. We conclude that under the relatively low light conditions and two-hour exposure duration used here, melanopsin activation is insufficient to produce any significant circadian phase advance. Moreover, it follows that the substantial phase advance produced by the blue light equated in melanopsin effectiveness to the white light is the result of activation of the color-opponent circuitry, not melanopsin, as commonly assumed. The implication of our result reported here is that since modest illumination level (ca. 500 lux) white lights presented for relatively short duration exposures (≤ 2 hours) are ineffective in stimulating melanopsin sufficiently to produce a phase advance, any practical indoor lighting solution to social jet lag and other problems associated with a delayed circadian clock should focus on stimulating the color opponent inputs to ipRGCs.

Previously, one hour of bright white (^~^10,000 lux) light produced a 40 minute advance in circadian phase^17^. When white lights are sufficiently bright, they can produce a phase advance by activating the much less sensitive melanopsin expressed in human ipRGCs compared to the 500-lux static white light that was ineffective here (Figure 2D). However, light that strongly modulates the S-cones for two hours (500 lux X 2 hr vs. ^~^10,000 Lux X 1 hr) amounts to 10X fewer lux-hours but produced a circadian phase advance per exposure hour that was twice as great. Thus, the S-cone modulating light is twice as effective as very bright white light at 1/20^th^ the intensity.

As a different alternative to static illumination, Zeitzer et al. administered 60 2-msec pulses of 473 Lux broad spectral band light over an hour and produced a phase change nearly half that of 1-hour 10,000 Lux static white light^18^. We assume that the increased effectiveness is due to the involvement of cone circuits, as in the experiments reported here, since transient white flashes drive spectrally opponent cone inputs to ipRGCs by virtue of differences in the temporal properties of their components. However, because of the spectrally opponent nature of the cone inputs to ipRGCs, modulating S- vs. LM cones is superior to non-spectrally selective cone modulation. The S-cone modulating light is 4 times more effective and the exchange between long and short wavelength components can be invisible whereas bright flashes every minute are not a practical alternative to traditional illumination.

Earlier, Spitschan and colleagues^19^ measured melatonin suppression using two light stimuli which differed exclusively in the amount of S-cone excitation by almost two orders of magnitude, but not in the excitation L and M cones, rods, and melanopsin. Since the light with stronger S-cone excitation did not differentially suppress melatonin, it might be interpreted to suggest a lack of support for a role for S-cone signals in circadian phototransduction. However, the Spitschan et al. experiment relies on the assumption of additivity which doesn’t apply to color opponent systems. Static white lights can produce strong S-cone excitation but provide zero drive to ipRGCs because of the opponent nature of the cone inputs. The “S cone isolating light” used by Spitschan was a pinkish color compared to “S- light” which was orangish. This is because to equate the two lights for L and M effectiveness the S+ light had to include about equal amounts of long and short wavelength light, nulling the color opponent response like what occurs with the white light, as illustrated in Figure 1D. Thus, the results reported here are consistent with those of Spitschan et al. showing that lights with strong S-cone excitation (a white light in our case and a pink one for Spitschan et al.) that balance S and (L+M) activation don’t have strong effects. In addition, our results are consistent with more recent results using narrowband lights which show that color opponent circuitry is involved in circadian phototransduction^10,11^.

The color of the sky at sunrise and sunset (Figure 1C) is the ideal cue for synchronizing one’s internal body clock to solar time. The intensity of light overhead can vary greatly for many reasons making it an unreliable indicator of the time of day, but the orange color of the sky at the horizon always indicates that it is sunrise or sunset. Retinal ganglion cells act as feature detectors. The color opponent inputs to ipRGCs confer the ability to act as sunrise/sunset detectors. The orange color of the horizon that characterizes the rising and setting sun produces a color contrast with the blue sky (Figure 1C). The blue and orange parts of the image on the retina produced by the sunset moving across the receptive field of an ipRGC activates the transient color-opponent response very strongly. As shown in Figure 1A, when our internal clock is aligned with solar time, sunrise occurs after the peak of the phase advance portion of the phase response curve and sunset occurs before the peak of the delayed phase portion. When the ipRGCs are strongly stimulated at both dawn and dusk the human phase response curve is perfectly tuned to keep the phase of our internal pacemaker precisely aligned with solar time.

Color opponent mechanisms are associated with sensory systems that regulate circadian activity throughout the animal kingdom including fish and reptiles^20,21^. Ancient single-celled organisms exhibit color sensitivity that they use to their circadian activity^22^. It appears that the capacity to sense colors originally evolved to serve circadian rhythms, not for hue perception^23^. The fact that primates have evolved multiple independent circuits that provide color-opponent inputs to ipRGCs is a testament to the importance of these sunrise and sunset detectors to our evolutionary survival. Thus, it makes perfect sense to develop lighting to use these color vision circuits to take control of our circadian wellbeing.

Our goal is to take control of our circadian rhythms by adding light exposures that strongly modulate Scone opponency in the morning in the context of the light experience in people’s regular daily lives. Thus, here, each subject was exposed to the experimental lights on a background of their regular daily lives as academics at the University of Washington. In this context, exposure to a 500-lux static white produced no significant phase advance but a light with the same melanopsin effectiveness that temporarily modulated S-cone color opponent circuitry produced phase advances, that if administered in the context of a person’s normal lighting routine, would be capable of offsetting the average 2.8-hour delay, therefore eliminating social jet lag.

The discoveries of color vision circuitry inputs to primate ipRGCs^7,8^ together with the evidence which has accumulated showing the role that circuitry in circadian phototransduction, indicate a complete paradigm shift in the strategy to develop healthy circadian lighting away from focusing on melanopsin to emphasizing the cone inputs. Melanopsin might have been emphasized over the powerful effects of the color-opponent inputs to ipRGCs because ideas about resetting of phase in humans have been extrapolated from experiments on rodents that have emphasized melanopsin. While it has been recognized that ipRGCs could be activated by classic photoreceptor input in the absence of melanopsin in mice^24^, neither M1 or M2 ipRGCs in mice were reported to have inputs from the color-opponent circuitry observed in primates;^25,26^ however, more recently, differential input between S and M cones were shown to produce responses in the suprachiasmatic nucleus of mice, recognizing the importance of cone inputs for circadian entrainment, especially in cone dominated species^27^. Here, we demonstrate that rather than focusing on melanopsin, under the constraints of making lights that appear white with intensities like standard artificial lighting used indoors, stimulating ipRGCs by modulating S-cones has promise to give people control of their circadian rhythms to improve mood, sleep, and health.

## Methods

All methods were performed in accordance with the relevant guidelines and regulations. Data collected and used in this study is available upon request.

### Miniature, programmable, and portable ganzfeld design

**Figure 3.**
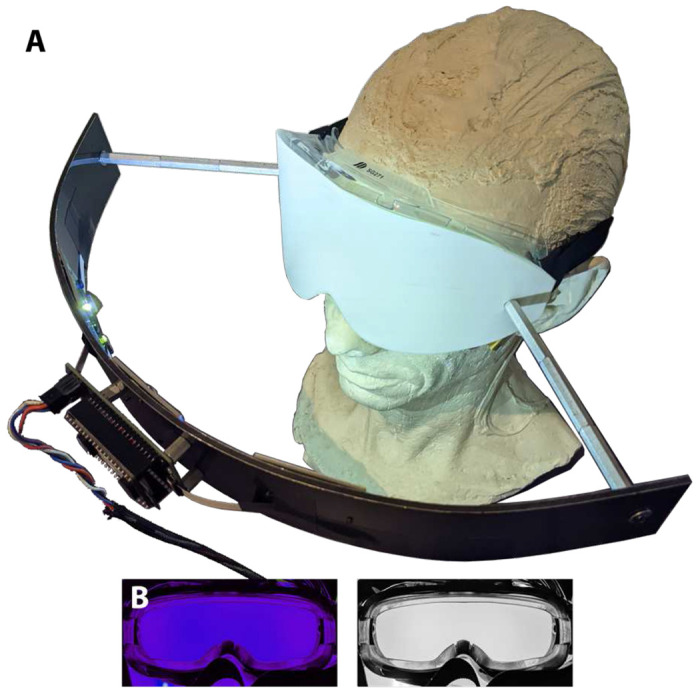
Battery powered portable “ganzfeld” light stimulator with self-contained uniform four color LED illumination programmable in intensity, temporal and spectral characteristics. **B.** The front diffuser of the illumination system goggles uniformly illuminated by the 476 nm LEDs (left) and static white (right).

Modified safety goggles were fitted with diffusers and LED illumination to provide the light stimuli (Figure 3). LEDs and LED driver circuitry were mounted to curved plastic-corrugated aluminum bands which were, in turn, mounted to the googles by metal standoffs (Figure 3). A spectrally flat transmissive diffuser (Lee filters, LEELux #400RW) replaced the original lens of the goggles. Three sets of four high powered LEDs (Luxeon CZ line by Lumileds) were mounted in each goggle stimulator, L1CU-VLT1 with peak at 426 nm, L1CU-BLU1 with peak at 476 nm, L1CU-LME1 with peak at 539 nm, and L1CU-RED1 with peak at 637 nm. Each had a continuous forward DC current rating of 350 mA and a 120-degree emission angle. Three pads for each of the 4 different LEDs were placed at regular intervals across the curved aluminum band with the middle pad positioned at the center of the goggle. This arrangement provided diffuse homogenous full-field illumination (Figure 3) covering approximately 130 degrees of visual angle.

The goggles illumination was controlled by custom made electronic constant current Pulse Width Modulation (PWM) control driver circuitry. This device was configured to allow LED settings to be stored on an EEPROM. These devices were calibrated and programmed in the laboratory and sent home with individual subjects. The spectral characteristics of the light reaching the eyes were measured using an CS-2000A spectroradiometer (Konica Minolta) positioned 1 meter behind each goggle. The two spectrums that were alternated temporally to drive high S-cone modulation were calculated theoretically using retinal sensitivities for S-, melanopsin, M-, and L-retinal sensitivities given by a photopigment template^28^ with peaks set at 420 nm, 480 nm, 530 nm, and 559 nm, respectively, corrected for absorption by the lens^29^. For the S-cone modulating light, the ratio of S-cone activation between the temporally alternated spectrums was 100:1, while L- and M-cone activations were held constant between the two temporal phases. The alternating spectrums (Figure 1D right; top and bottom) were programmed onto the goggles and modulated at 19 Hz presented as a square wave with 50% duty cycle. The radiance of these lights measured at the back of the goggles was 150.5 μW/cm^2^. The alternation of the two spectrums produced approximately 500 lux at the subject’s pupil plane as measured with a lux meter (Digital Light Meter, LX 1330B). Melanopsin activation was determined by integrating the measured time averaged spectrum with the corneal sensitivity for melanopsin. The two other conditions, the static white light spectrum (Figure 1A) which produce a radiance measured at the back of the goggles of 72.9 μW/cm^2^ and the static blue spectrum from the 476 nm LED (Figure 1B) which produce a radiance measured at the back of the goggles of 31.6 μW/cm^2^, were adjusted in intensity to produce the same time averaged melanopsin activation as the Scone modulated light.

### Human Subjects

The Institutional Review Board at the University of Washington approved the human subject’s research. Research involving human subjects was performed in accordance with local and federal regulations. Human subjects research adhered to the principles embodied in the Declaration of Helsinki. Informed consent was obtained from all participants. The subjects were adult volunteers from the University of Washington community in Seattle.

Six healthy adult (2 male and 4 female) subjects (mean age = 30; range 23-43) continued with their daily academic lives during the winter months (December - February) in Seattle, WA over the course of the experiments. The purpose of the experiments was to determine the effects on circadian phase of three different lighting paradigms which were viewed for two hours centered 10.5 hours after their individual DLMO. Lights administered at this time should produce the maximum circadian phase advance (Figure 1A). Circadian phase was determined from the rise in evening melatonin levels assayed from saliva samples. To measure phase accurately it was important to identify subjects with a robust, reliable evening rise in salivary melatonin. In addition, it is important that our participants are stability entrained to the 24-hour environmental cycle even though we expect most members of the University of Washington university community to suffer from some amount of phase delay. New recruits collected baseline evening salivary melatonin samples every hour from 6 PM until 2 PM. During this period, they were instructed to generally keep illumination levels as measured by an illuminometer below 10 lux. Short periods of higher illumination were allowed, when necessary, but were always kept below 30 lux. Subjects also confirmed that they were keeping a regular sleep-wake schedule in the days surrounding the experiment. After the first baseline salivary melatonin measurement, the only participants that continued with the experiment were those that showed a robust rise in salivary melatonin between 6 pm to 2 am. Four of the original recruits did not meet this requirement. Failure may be because subjects’ internal clocks are free running, or they may be arrhythmic. This high number of failures may be a consequence of the large number of gray and short winter days in Seattle.

Of the six subjects who met the inclusion criteria, all are graduate students, post-docs and one assistant professor involved in studies related to circadian rhythms and five of them are co-authors on this manuscript. As such, they were all very motivated to adhere to the somewhat grueling demands of the protocol. These included adhering to the strict evening lighting regimen, collecting saliva on a strict schedule, proper handling of the saliva samples and viewing the lights at the times and durations specified. We believe that having motivated compliant, participants was a key to obtaining precise and reliable results. Salivary melatonin measurements are objective so the fact that participants were not naive to the objectives of the experiment could not bias the results.

### Experimental protocol for viewing light stimuli

The experiment was conducted during the COVID19 pandemic. Safety protocols prevented participants from coming to the laboratory for experimental procedures, thus, all experiment procedures were conducted in participants’ homes. Saliva samples were collected by the subjects at one-hour intervals starting at 6 PM PST and placed on dry ice immediately after collection. Two separate saliva samples were collected at each time point, which were analyzed separately and averaged to minimize noise for each individual timepoint. Since the experiments were done in the winter in Seattle, saliva collection was done well after sunset so there was no possibility of exposure to sunlight during saliva collection and subjects stayed in their homes with the illumination generally kept below 10 lux and always below 30 lux. Circadian timing was measured by the dim light salivary melatonin onset (DLMO, Salimetrics melatonin ELISA). DLMO_20%_ was calculated as the time point at which melatonin levels reached 20% of the fitted peak-to-trough amplitude of each person’s data. The data was fitted to an integrated Gaussian (error function) by minimizing the sum of least squares. Maximum phase advances were assumed to occur 10.5 hours after DLMO_20%_. Administrations of a 2-hour light pulse of the therapeutic lights were therefore centered around 10.5 hours after DLMO_20%_. Lights were administered in the subjects homes the morning after the baseline internal circadian timing was measured. To determine the phase advance caused by each light, circadian timing was remeasured the evening of the day the light was administered. Phase advances were calculated as the difference between DLMO_20%_ after light administration and baseline DLMO_20%_. Differences in phase produced by the light treatments were evaluated using a paired t-test using each person DLMO measurement before and after treatment as a pair.

## Data Availability

Contact J.A.K. to request the data from this study.
